# Design and Implementation of a Cloud PACS Architecture

**DOI:** 10.3390/s22218569

**Published:** 2022-11-07

**Authors:** Jacek Kawa, Bartłomiej Pyciński, Michał Smoliński, Paweł Bożek, Marek Kwasecki, Bartosz Pietrzyk, Dariusz Szymański

**Affiliations:** 1 Radpoint Sp. z o.o., Ceglana 35, 40-514 Katowice, Poland; 2 Faculty of Biomedical Engineering, Silesian University of Technology, Roosevelta 40, 41-800 Zabrze, Poland; 3 Department of Radiology and Radiodiagnostics in Zabrze, Medical University of Silesia in Katowice, 3 Maja 13/15, 41-800 Zabrze, Poland

**Keywords:** cloud, cloud storage, picture archiving and communication system, vendor neutral archive, medical imaging

## Abstract

The limitations of the classic PACS (picture archiving and communication system), such as the backward-compatible DICOM network architecture and poor security and maintenance, are well-known. They are challenged by various existing solutions employing cloud-related patterns and services. However, a full-scale cloud-native PACS has not yet been demonstrated. The paper introduces a vendor-neutral cloud PACS architecture. It is divided into two main components: a cloud platform and an access device. The cloud platform is responsible for nearline (long-term) image archive, data flow, and backend management. It operates in multi-tenant mode. The access device is responsible for the local DICOM (Digital Imaging and Communications in Medicine) interface and serves as a gateway to cloud services. The cloud PACS was first implemented in an Amazon Web Services environment. It employs a number of general-purpose services designed or adapted for a cloud environment, including Kafka, OpenSearch, and Memcached. Custom services, such as a central PACS node, queue manager, or flow worker, also developed as cloud microservices, bring DICOM support, external integration, and a management layer. The PACS was verified using image traffic from, among others, computed tomography (CT), magnetic resonance (MR), and computed radiography (CR) modalities. During the test, the system was reliably storing and accessing image data. In following tests, scaling behavior differences between the monolithic Dcm4chee server and the proposed solution are shown. The growing number of parallel connections did not influence the monolithic server’s overall throughput, whereas the performance of cloud PACS noticeably increased. In the final test, different retrieval patterns were evaluated to assess performance under different scenarios. The current production environment stores over 450 TB of image data and handles over 4000 DICOM nodes.

## 1. Introduction

Picture archiving and communication systems (PACSs) are the main technological infrastructure enabling the management of medical data [1]. The traditional functions of a PACS are acquisition, storage, distribution, and display of images [2]. However, nowadays, the PACS term often refers to the generic storage and distribution part that devices are connected to [3], sometimes interchangeably with the vendor neutral archive (VNA) [4], with the latter emphasizing interoperability and the storage of non-image data. In this paper, PACS and VNA both refer to the infrastructure for storage and communication of medical data coming from DICOM compliant devices and applications.

Origins of standardization in the PACS field go back to the 1980s, when the American College of Radiology (ACR) and the National Electrical Manufacturers Association (NEMA) developed standards for the interconnection of digital imaging devices [5]. Since then, the standards have evolved, but backward compatibility has always been essential. Version 3.0 of ACR-NEMA, referred to as DICOM (Digital Imaging and Communications in Medicine), and dated to 1993, has been widely adopted in all radiological computer systems [6]. However, nowadays, DICOM archives include also image and signal data generated by other departments, including hematology, laryngology, dermatology [7], cardiology [8], pathology [9], oncology [10], and urology [11]. Standardization work is ongoing in the field of neurophysiological signals [12]. DICOM can also be used to store proprietary formats, such as FIB–SEM microscope scanners [13].

The DICOM standard was defined when the internet was in its early development. In the contemporary network perspective, the original DICOM solutions do not fit the current requirements of security or efficiency. Although the standard is kept up to date with modern solutions (e.g., new imaging modalities, secure socket layer (SSL) communication, representational state transfer (REST), etc.), its main profound advantage, i.e., backward compatibility, is also a significant limitation for PACS developers [14].

The best practice nowadays is to isolate the local imaging center network physically or on logical level, and filter incoming and outgoing traffic [15]. VPNs (virtual private networks) bridging local segments should be employed when image sharing between locations is necessary. However, long round-trip times make DICOM less efficient in distributed networks, and hybrid solutions are harder to maintain. On the other hand, PACS systems open to the world due to misconfiguration or poor design are surprisingly common [16].

The other challenge for PACS comes from the amount of archived data. Preserving at least two copies of images or other data within the system is one of the traditional requirements [2], maintained by selecting appropriate flow and retention rules. However, keeping the growing amount of medical data as regulated by national laws requires constant hardware and software upgrades. Long-term backup may also be maintained by girding systems located in different physical locations [17], or by selecting external storage [18].

There are some non-technical issues to address as well. Deployment of PACS requires considerable financial resources that must be allocated for the hardware and software, as well as for support and training [19]. Systems are not flexible and force staff to adapt to the system requirements [20] or to develop in-house solutions [21]. Moreover, a number of small healthcare institutions, notably employing ultrasound imaging, do not connect their equipment to PACS [22] or archive data at all [23].

Consequently, the necessity to switch to a modern distributed system architecture is predicted. Cloud computing, featuring such concepts as software and hardware as a service, is a logical next step [24].

### 1.1. Evolution of Cloud PACS

Early works predicted that moving to the cloud may mitigate hardware overprovisioning and underuse [25]. Data centers make scaling up (hardware upgrade) feasible. They provide flexible virtual-machine configuration, efficient internal and external networking, and additional services such as external backup or distributing the virtual machines among different physical locations [26]. The related problems of data integrity and transfer security were addressed in [27,28]. Several studies discussed key aspects of when a PACS migration or upgrade has to be performed [19,29,30,31,32].

However, early practical solutions were typically straightforward HTTP front-ends facilitating the communication (see, for example, a work of Valente et al. [33]). However, in 2017, Álvarez et al. described a solution [34] where a storage back-end could be provided by any cloud-based system with a sufficient API to communicate, allowing an external backup or image repository.

In 2018, Sébastien Jodogne et al. presented Orthanc [35], the initial releases of which date back to 2012. The original set of functions included a REST interface for traditional PACS. It enabled external software, e.g., a computer-aided diagnosis module, to communicate with DICOM-only PACS. With time, the system evolved to include a full DICOM interface, basic worklist management, DICOM web support, and additional toolkits, and is currently the gold standard of external interface for modern PACS systems. Orthanc as a VNA provides a powerful environment to automate and optimize the management of medical images. The system has been implemented in numerous radiological departments and hospitals worldwide. It is employed as a PACS engine, as well as software that integrates algorithms of artificial intelligence with radiological data [36]. However, despite employing cloud design patterns, it is not well suited to be used as a microservice in a cloud-native environment.

Dcm4chee is an archive and image manager that has been used widely for the last 20 years [37]. Its architecture consists of multiple modules allowing users to customize the system. Data flow may be configured to store imaging data in online or nearline storage. The commonly used 2.x series [37] provides a monolithic solution. The more recent 5.x series, introduced in 2015, may be directly deployed in a cloud environment and use some cloud-native services (e.g., S3 storage). However, due to its memory footprint and resource utilization, the Dcm4chee can hardly be regarded as microservice and a cloud-native service itself.

Another growing PACS solution worth mentioning in this context is Dicoogle [38]. It started as an indexing front-end for a dcm4chee server, enabling faster retrieval using cloud services [39]; then, it was gradually developed into a cloud system featuring plugin-based architecture [40]. Later, the system was employed as a proof of concept PACS, where data are stored in a distributed cluster using a peer-to-peer protocol [41]. Dicoogle has many cloud-native technologies, as described further in this section, yet lacks some production-grade features, e.g., multi-tenant capability.

A few more PACS systems designs were presented in [42]. The advantages and disadvantages of various PACS systems, as well as the presented solution, are summarized in Table 1. Moreover, there are some commercial PACS available that advertise a cloud environment (e.g., Omega AI VNA delivered by Ramsoft, Toronto, Canada), yet no technical details of the solutions are provided.

Moreover, it is worth pointing out that several attempts to describe AI-based medical imaging services and their deployment in hospitals have been made in past years [43,44], as well as recently [31,45,46,47]. An interesting description of the opportunities and challenges of artificial intelligence in the medical field was presented by L. Jiang et al. [48]. However, AI services do not need to maintain backward compatibility with legacy hardware and are easier to upgrade to modern architectures.

Briefly summarizing, the number of solutions deployed for the cloud environment or employing cloud technologies is increasing. However, a full-scale cloud PACS developed as a cloud-native solution has not been demonstrated yet.

### 1.2. Cloud-Native Systems

The term ‘cloud native’ is used to distinguish between systems migrated to the cloud and those designed for the cloud. It is seldom defined beyond that, yet typically associated with five properties [49]: global-scale operation, good scaling with a growing number of users, built on the assumption that infrastructure failure at some point is inevitable, seamless and uninterruptible upgrade is possible, and security is built-in. One of the basic design patterns is microservice usage. Microservices are [50] an architectural approach meant to improve scalability, agility, and autonomy. They assume self-management and light weight. They are expected to be independent and responsible for single functions [51]. Microservice-based systems can take full advantage of the cloud-delivery model [52]. Another essential cloud-native pattern is employing message brokers to provide loose microservice communication [11]. Different message delivery models of the available brokers (e.g., at-least-once, at-most-once) provide desired properties for the system and enable robustness in case of failure. Independence from the specific cloud vendor (cloud vendor agnosticism) in the form of additional abstraction (e.g., federated storage, stateless operation) is also an important factor in today’s cloud-native design [53], permitting migration between providers without loss of functionality.

Compared with on-premise or cloud-migrated solutions, the advantages of cloud-native design are [54]: performance coming from employing the native services, efficiency in the use of underlying resources, cost reduction coming mainly from the said increased efficiency, and scalability. These can directly benefit the PACS applications.

With the cloud-native approach, one can provide a PACS that scales equally well to the needs of a radiological department and to a small physician’s practice equipped with a single ultrasound machine. Consequently, the deployment and maintenance costs can be proportional to the generated load, not to the number of installation sites. Cloud-native services can be employed to provide fault tolerance and high availability (HA). Moreover, the cloud-native approach is, by design, service-oriented and can flexibly accommodate additional features, such as healthcare integration (e.g., Health Level Seven (HL7) router), external computer-aided diagnosis modules (automatic routing of selected studies to various providers), or GPU acceleration.

The long-term goal of this study was to develop a highly available (HA) cloud PACS/VNA system featuring shared-nothing cloud-native architecture based on stateless microservices, message bus, federated storage, and agent devices integrating local network segments operating DICOM. The state-of-the-art solutions were to secure external (accessible from the internet) interfaces. The operational cost was meant to be affordable for most end-users and related to the amount of generated traffic and storage.

Designing scalable infrastructure was seen as crucial for the efficient provisioning of high-availability services. DICOM (image or video) traffic may rapidly change by magnitude. The selected, shared-nothing approach implies the independence of the microservices and facilitates dynamic operation. Basing the solution on the web stack was argued to be a straightforward way to provide easy and secure access for web and mobile agents.

Infrastructure was meant to be available for different clients, with the traffic and data securely separated. Online and nearline (or offline) storage were to be defined based on the local devices and cloud services. Configuration of the PACS was meant to be accessible for non-trained IT specialists, with low-level options reasonably pre-configured and local devices auto-provisioned to receive DICOM traffic.

In order to meet the presented requirements, a software architecture was developed and a system was implemented.

In this paper, a novel cloud-native PACS architecture is presented together with a working, original system designed on its basis. The system employs a number of general-purpose services designed or adapted for a cloud environment, such as Kafka, OpenSearch, or Memcached. Custom services, such as Central PACS Node and Queue Manager of Flow Worker, also developed as cloud microservices, bring DICOM support, external integration, and a management layer. All services are deployed and integrated up to contemporary cloud-native standards.

### 1.3. Contribution

The novelty of this work is the concept development and implementation of a fully functional cloud-native PACS/VNA system. It follows cloud principles and design patterns, including shared-nothing architecture, microservices, message bus, and containerization. The provided PACS has proven its usability and stability, handling more than four thousand DICOM nodes. To the authors’ knowledge, the presented system is the first full-scale PACS explicitly designed for cloud usage.

## 2. Materials and Methods

**Figure 1:**
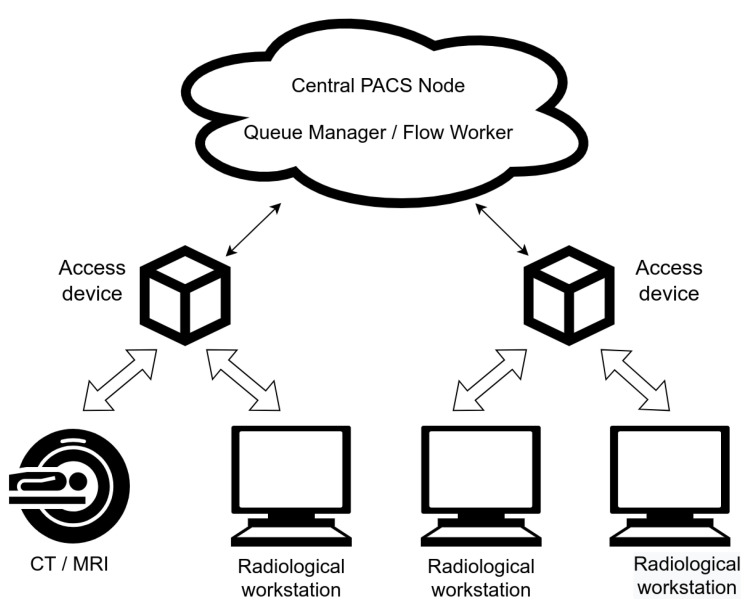
PACS diagram (schematic).

**Figure 2:**
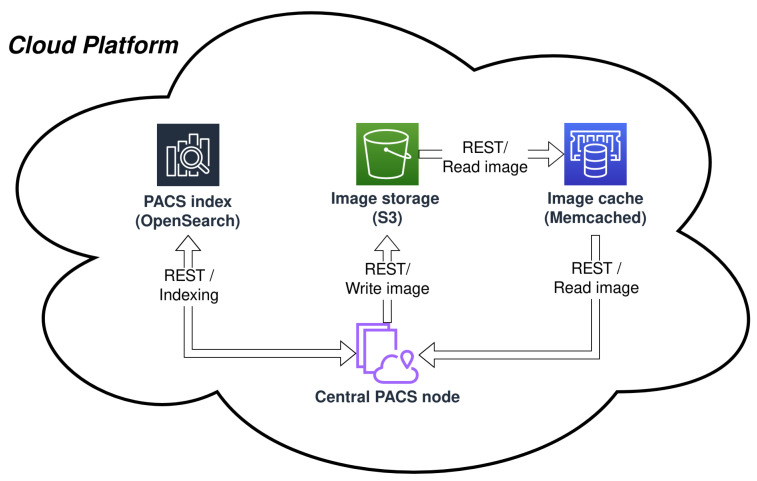
Internal elements of the central PACS node.

**Figure 3:**
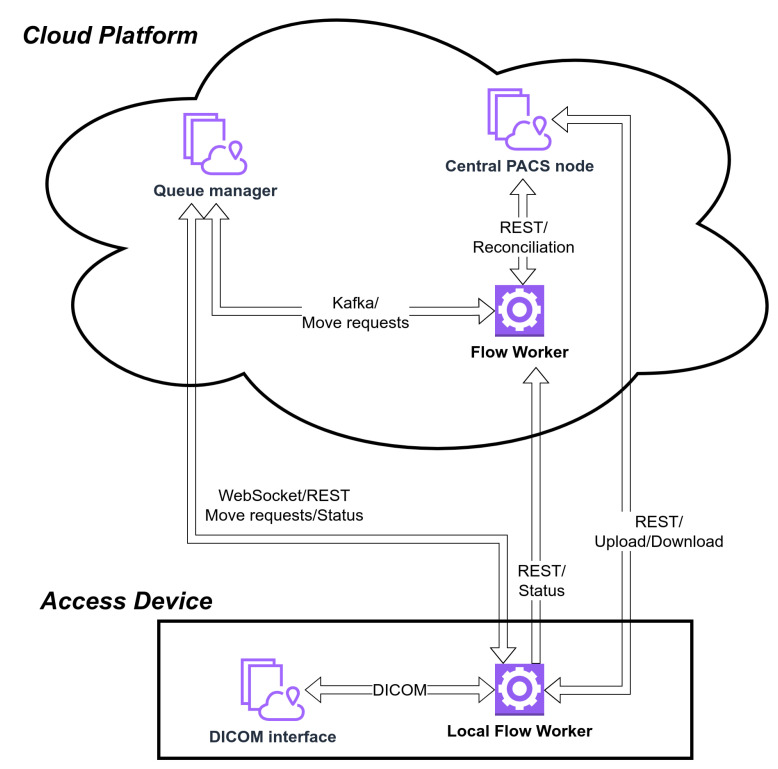
Connections between the access device and the cloud platform.

The discussed system consists of four main elements (Figure 1). The core is a central PACS node, which is a cloud service responsible for image storage, search, and retrieval. The second part consists of a queue manager and a flow worker—these are components responsible for managing the transfers within the cloud environment and between cloud and access devices. Access devices are the third element. They host local, online DICOM storage, and local instances of the flow worker. Access devices are responsible for carrying out the queue-management tasks retrieved from the cloud counterpart. The last element of the system is composed of various supporting services responsible for authentication, proxying, provisioning, monitoring, etc. These services were omitted for brevity in Figure 1, Figure 2 and Figure 3.

All the key elements are designed as stateless microservices operating in a shared-nothing environment. The business flow is coordinated via asynchronous message bus (Kafka) with an at-least-once delivery guarantee. Microservices employ dual REST and GRPC communication for synchronous backend communication.

The four parts of systems are described in the following four sections, respectively.

### 2.1. Central PACS Node

The central PACS node is a custom, lightweight server natively implementing several APIs (application programming interface):

- DICOM, DIMSE (DICOM message service element)-based interface, featuring a set of basic C-STORE, C-FIND, C-MOVE operations;
- Native DICOMweb interface, featuring STOW-RS, QIDO-RS, WADO-RS—web services providing access to storing, querying, and retrieving DICOM objects;
- Custom REST (Representational state transfer) API providing additional capabilities.

The central PACS node is designed to directly utilize the AWS S3 storage service as an image archive. File storage is hash-keyed and accessed on the instance level. Memcached is employed as a caching service, and the cache is populated with the series or study data on the instance access events (Figure 2).

Indexing in OpenSearch (formerly Elasticsearch) is performed on various levels to speed up typical usage patterns. All DICOM tags on the instance and series level are available for use in search queries through DICOM as well as DICOMweb and REST interfaces. It is worth mentioning that the subset of indexed DICOM tags supersedes that of a typical monolithic PACS.

The central PACS node is designed as a stateless microservice. All the information necessary to process the query is directly provided within the request or retrieved from the invocation context. The service is multi-tenant ready, which means that several S3, OpenSearch databases, Memcached instances, etc., might be handled simultaneously.

In the system, the service is containerized with the Docker system and clustered with a Docker swarm. The REST interface is used for internal operations, while the DICOM and DICOMweb interfaces are provided for occasional use by road warrior-like radiologists, connecting PACS with wireguard VPNs, and for future use with external AI CAD (artificial intelligence computer-aided diagnosis) modules.

The primary consumers of the central PACS node interface are the queue manager and flow workers.

### 2.2. Queue Manager and Flow Workers

**Figure 4:**
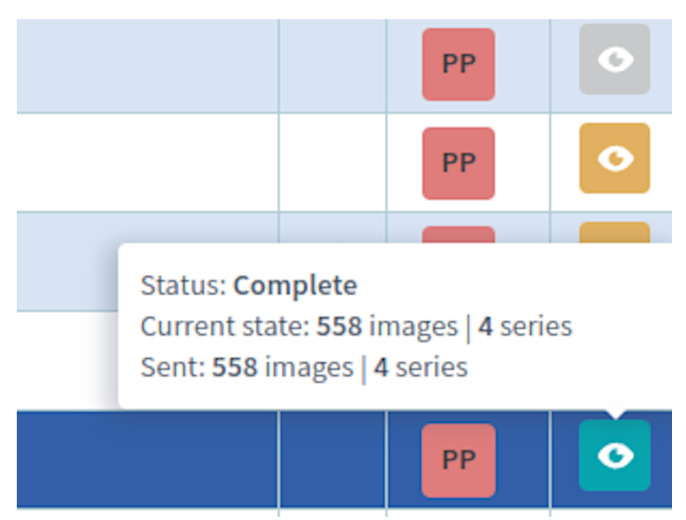
Internal queue state and priority visualization (partial screenshot). Green status is assigned to studies fully available in Central PACS, orange to studies during transfer, and gray marks for cases waiting for transfer. PP denotes the highest priority.

The queue manager is a service responsible for internal and external transfers within the system (Figure 3). The transfer requests are carried out by flow workers (within the cloud environment, e.g., between archives) or local flow workers (described in the following section). The service supports transfer priorities and constantly monitors the execution of tasks (Figure 4).

Internally, the queue manager service and flow workers are lightweight modules developed in Node.js, and containerized. The transfer requests are delivered via the system message bus (Kafka) and are handled by the first available worker concerning priorities.

### 2.3. Access Devices

Access devices are PC-grade microcomputers featuring an SSD drive, two 1GE network interfaces (LAN and WAN), and hardware security acceleration (AES-NI CPU capability).

**Figure 5:**
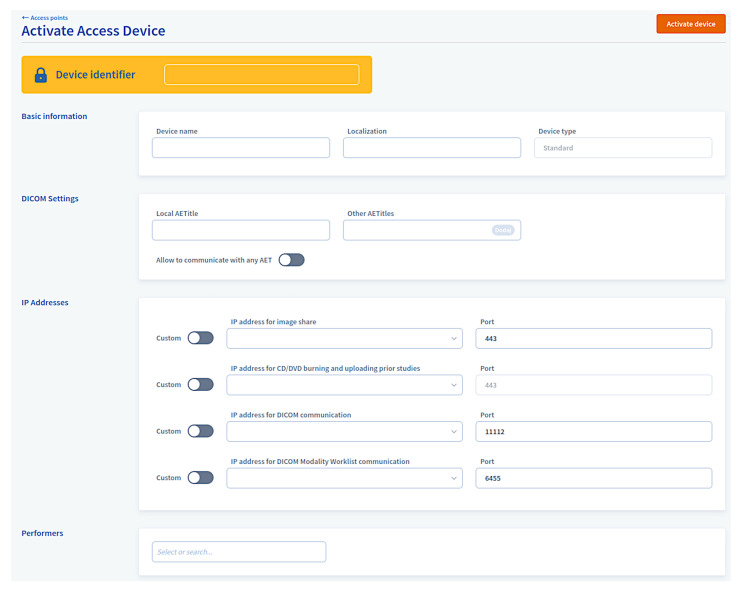
Access device activation.

The devices are activated in the PACS system (Figure 5) based on the hardware fingerprint and administrator action and—once authorized—are provisioned with the connection data. The platform’s internal interfaces are secured from the outside by access tokens and gateway proxy.

The activated access device connects to the platform, opens the WebSocket channel, and awaits requests. Local events are forwarded to the platform using a separate REST channel.

The behavior of the access device and communication channel are constantly monitored, and dedicated platform services maintain the health level.

#### 2.3.1. DICOM Interface

The DICOM interface service is a monolithic DICOM server remotely configured via the access device management services.

The configuration, including the local network topology (DICOM nodes), is provisioned from the central platform repository and managed via the platform’s graphical user interface.

The subset of DICOM services supported by the server is compliant with IHE (Integrating the Healthcare Enterprise) profiles.

Events generated on the DICOM interface are forwarded to the cloud queue manager and, if necessary, the transfer action to and from the access device.

#### 2.3.2. Local Flow Worker

The local flow worker is responsible for executing cloud queue manager requests on the access device site. The local flow worker retrieves requests via WebSocket channels and uses the REST interface to connect to internal cloud services to store or retrieve images.

### 2.4. Supporting Services

Supporting services include various auxiliary software components, such as:

- Single Sign On, responsible for credential validation and JWT token distribution;
- Proxy layer, responsible for authentication and redirection of external requests. For example, the proxy layer permits access device communication with internal cloud services;
- The provisioning module monitors access devices’ state and deploys updates.

The system is monitored by a custom stack of [Sentry]–Prometheus–Grafana and provider’s services (CloudWatch). Additional metrics are produced for use in scaling procedures.

## 3. Data Flow

In the current section, typical usage communication scenarios are depicted. The acquisition device (CT scanner) and the radiological workstation represent the user site connected to the access device.

### 3.1. CT to Cloud

A series of instances are first acquired and stored in the CT system. Once the series acquisition is completed, all the instances are one-by-one transmitted using C-STORE to the DICOM interface of the access device located within the same LAN segment. The retrieval of an instance generates the event, which is forwarded to the cloud queue manager. The manager selects the optimal time (e.g., lack of high-priority traffic) to issue a cloud transfer request that the local flow worker subsequently carries in the access device. As a result, the central PACS node retrieves the DICOM instance, indexes it, and updates cloud storage.

### 3.2. Local Archive to the Workstation

In this scenario, image instances requested by the local user are already available in the online archive of the access device (e.g., available from the previous transfers or sent to a device as a result of autorouting or prefetching). C-MOVE or WADO-RS DICOM services are directly employed.

### 3.3. Cloud to Workstation

In this scenario, the client requests image instances available only at the cloud site. In such a case, the results are marked as nearline, and the background queue manager activity is started to transfer the missing instances to the access device. Once they are locally available, C-MOVE is executed and the instances are locally stored.

### 3.4. Between PACS Nodes

Transfer between cloud-located archives may occur in case of, e.g., the application of the AI CAD modules. In such a case, the cloud flow worker performs the transfer task requested by the queue manager.

A road-warrior user accessing the platform is an additional common scenario. A user may do it directly, after authorization, via DICOMweb interface or by employing a VPN service to a jump-host virtual server run in a separate security zone of the cloud platform, enabling DICOM communication.

## 4. Results

The cloud PACS is currently in the production phase. The system’s state is summarized in Table 2. As of 30 June 2022, the system handles ca. 4600 DICOM nodes.

### 4.1. The Behavior of the System under Simulated Load

For the tests, a separate environment has been configured as follows:

- Four central PACS nodes have been set up on separate c5d.large AWS instances; on each host, the container network is bridged to the host network and the PACS node is the only microservice running; the Docker swarm system supervises the cluster;
- Eight cache hosts (Memcached) have been set up using r5a.large EC machines and connected to S3 storage;
- Elasticsearch (OpenSearch) index is initialized;
- Twenty access devices are connected to the system using a broadband connection (20–100 Mbps).

Traffic was redirected to the system via access devices, matching 117 different imaging device models (Table 3) acquiring and archiving images for seven consecutive days:

- Day 1: initial eight devices;
- Days 2–7: remaining devices.

**Figure 6:**
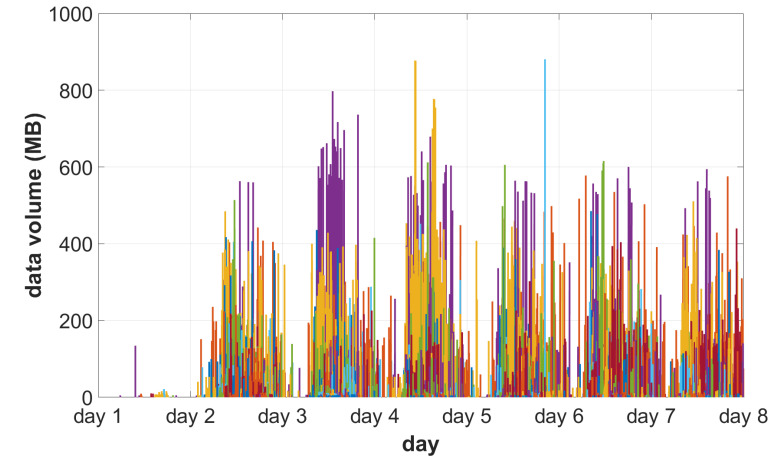
Traffic visualization during test days. Each series is marked with a separate bar. Different colors indicate acquisition devices.

**Figure 7:**
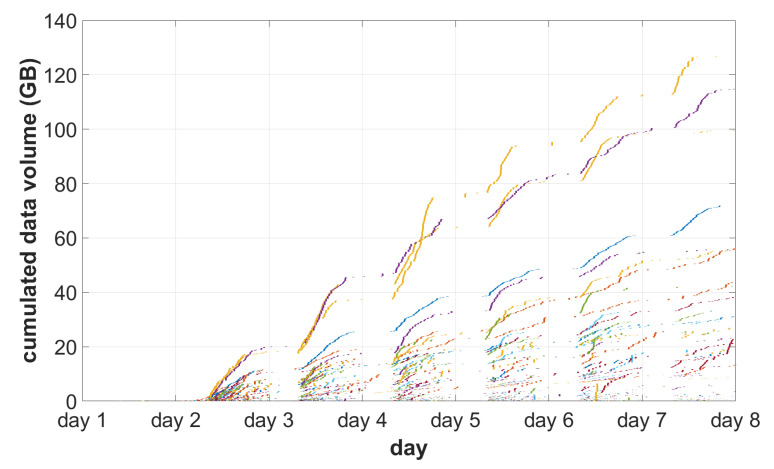
Cumulative traffic visualization during test days. Each imaging device’s model activity is composed of segments corresponding to a single series. The colors match Figure 6.

The traffic pattern [55] is visualized in Figure 6 and Figure 7. The former presents the size of each series transferred to a central system, while the latter shows how the volume of data is distributed among different acquisition devices.

The image stream was accepted without stalls and interruptions. A total 1.3 TB of data was stored in the system during the process. The acquired data was used afterward to tune scaling behavior.

The central PACS node was then evaluated in a randomized search using an extended, flat query with optional keys, i.e., all series matching the following criteria:

1. Created on each day of the test;
2. Created at 8 am.

**Figure 8:**
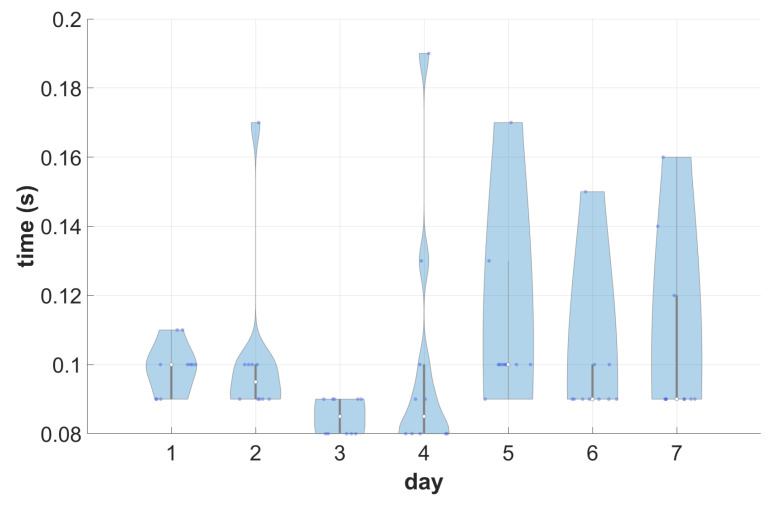
Searching all series created at a specific time. Total elapsed time was measured (since the issue of the request to display results).

A REST query was issued directly to the central PACS node from a separate virtual machine inside the same cloud environment and elapsed time was measured. The experiment was repeated ten times. The measured mean answer time was 0.099 s ± 0.02. Detailed results are visualized in Figure 8. In all but the last day-related case, the average time was under 0.1 s. However, there were certain queries that took up to twice as long.

**Figure 9:**
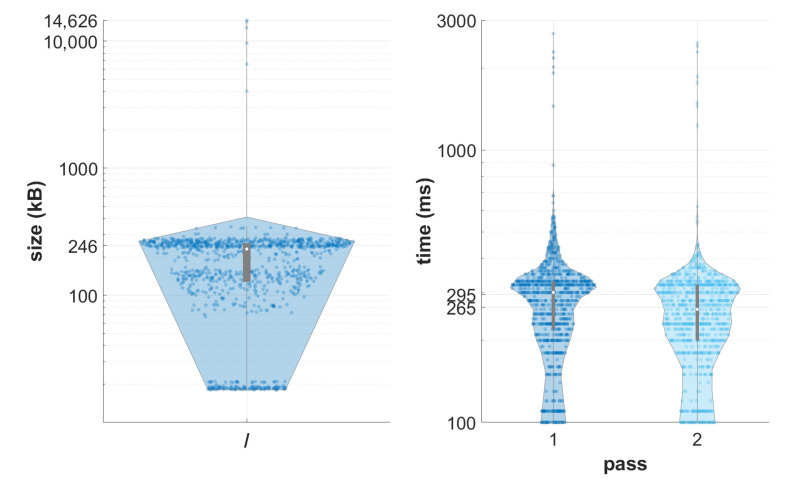
Data retrieval. The left part depicts the test data set; most instances occupied several hundred kB. The right part shows the total retrieval time (from issuing the request to finishing the write of the instance in the VM storage). Results are shown on a logarithmic scale for brevity.

In the next test, instance access time was evaluated. One thousand random identifiers were selected from a set containing 4874 identifiers with the same SeriesDate attribute (i.e., on average, one of every five instances of each series/study created within the same day was selected). Each instance was then retrieved using REST API (total 251 MB of data). The test was repeated twice (second pass after no less than 120 s, no other queries executed in between) with the assumption that during the second pass, some series might be cached by the caching engine. Results for both passes are presented in Figure 9. Compared to the initial pass, the average observed retrieval time in the second run was ca. 15% shorter.

### 4.2. Comparison of Data Retrieval and Search in the Monolithic and Cloud PACS

For the next tests, a different set-up was prepared in the AWS environment:

- A single, monolithic Dcm4chee 2.18.3 server was installed on an m5n.xlarge virtual instance (four logical processors, 16 GB RAM, Elastic Block Store (EBS) with 3.5 Gbps capacity and 25 Gbps bandwidth network). The server was configured to use the EBS storage local to the machine, external index in PostgreSQL database, accept and send 64 kB DICOM PDUs (protocol data units), and allow up to 128 simultaneous connections without a limit on underlying asynchronous operations;
- A cluster of two central PACS nodes was set up on two m5n.xlarge instances. Between nodes, traffic was balanced using the Application Load Balancer service on c6gn.large instance (two virtual processors, 10Gbps bandwidth network). A cluster was attached to the S3 storage and OpenSearch index. Central PACS nodes were configured to handle DICOM connections in a serial manner (e.g., in the case of C-MOVE operation, all instances were sent one after another). Four Memcached nodes were configured. 64 kB DICOM PDUs were allowed;
- A testing machine was configured. An m5n.large instance was configured with Ubuntu 20.04.5 system, equipped with dcmtk toolkit, and put under netdata service monitoring;
- A DICOM dataset was generated consisting of 500 studies of 10 patients. Each study consisted of five series with 104 uncompressed (Little Ending Explicit) CT instances embedding the same image content. The size of a single instance was ca. 202 kB, accounting for a volume of a single study being ca. 205 MB and the total volume of the dataset being 100 GB. The dataset was uploaded to both DICOM servers on the day preceding the testing.

During the initial test, a set of parallel C-MOVE operations was initiated from a testing machine. First, a number of dcmqrscp nodes were configured to accept incoming C-STORE requests and permit simultaneous (parallel) associations. Next, for each test iteration, a set of 80 previously unused studies was selected by the StudyInstanceUID identifier. Then, a number of parallel transfer workers were run. Each worker was assigned a matching number of studies to fetch from the tested server and was allowed to fetch them one by one (in serial). The worker employed dcmtk’s movescu application configured to operate on 64 kB PDUs. Transfers were evenly distributed to dcmqrscp instances. The central PACS node Memcache was flushed between attempts.

**Figure 10:**
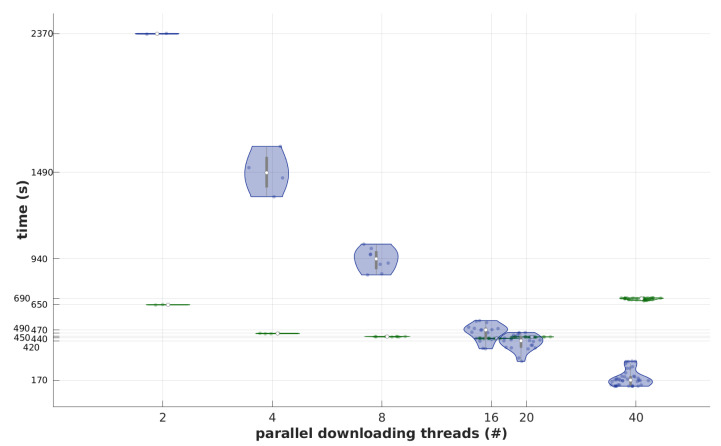
Image retrieval test set. On the horizontal axis, a number of parallel workers are depicted (each worker running in a separate thread). On the vertical axis, the time of every single worker in a batch, as well as the median time for each run, is presented. Green and blue boxes show times when retrieving from the Dcm4chee server or the cluster of central PACS nodes, respectively. In each run, the same amount (80) of studies were retrieved (ca. 16 GB). Most of the Dcm4chee-related operations finished in a similar time (hence the boxes are flattened), while the cluster-related operations were time-varying with average decreasing with the growing number of workers (throughput increases).

The results presented in Figure 10 show that in the presented system, performance grows with the increase of network connections, while the monolithic system does not benefit. However, in this particular configuration, the performance of the DICOM interface of the central PACS node for small workloads of the cloud solution was inferior to the monolithic.

As the next test, performance of a typical search operation performance was checked. A C-FIND query was issued ten times to monolithic and cloud solutions to find all instance identifiers related to a specified series, previously neither queried nor referenced. Measured times for Dcm4chee and central PACS node were 0.104 ± 0.061 s and 0.105 ± 0.018 s, respectively. Notably, the first query to the cloud solution was finished after 0.14 s, while the first query to the monolithic solution took 0.23 s.

### 4.3. Performance Analysis for Different Loads and Study Sizes

With the final test, the behavior of the cluster was analyzed for different loads and study sizes. A similar cluster configuration to that shown in the previous section was used. However, the number of Memcached nodes was reduced to two instances, and only the central PACS nodes were evaluated this time.

During the test, a DICOM dataset consisting of 320 studies was generated. Each study consisted of four series of uncompressed (Little Endian Explicit Transfer Syntax) 512 kB CT instances. The number of instances in series was selected to obtain 2 MB, 4 MB, 8 MB, …, 1024 MB study volumes, with 32 studies of each size. These studies were successively retrieved in four, eight, and sixteen parallel threads. Each time, the cache was dropped prior to the test run, and the same procedure was used to download the data. DICOM PDUs of 64 kB were employed.

The data retrieval results, presented in Table 4, show similar performance for each set-up, with slightly increased throughput for larger study sizes and greater variability for smaller study sizes. When doubling the number of threads, the performance doubles for the 32–128 MB study size. The occasional boost (i.e., more than doubling the performance) is attributed to background per-study read-ahead Memcached operation. For larger studies, the gain is lower (e.g., ca. 170% for 512 MB studies or 180% for 1024 MB studies). Similar observations also hold for different instance sizes. However, the performance generally decreases with the reduced size of the instance (e.g., 2.9 s–3.8 s per 100 instances for eight threads and 1024 MB study consisting of 256 kb instances).

## 5. Discussion

The presented system follows state-of-the-art practices for the cloud-native systems, such as stateless microservices or a message bus. High scalability was a must-have feature and was implemented using contemporary software tools. Moreover, to the authors’ knowledge, the presented system is the first PACS explicitly designed as a cloud-native solution.

As shown in Section 4.1, the system can handle multiple data streams. Under typical scenarios, DICOM data are buffered on at least a series level and sent in bursts. The proposed system can handle bursts in serial or parallel by accepting multiple simultaneous associations. In this aspect, it pairs with monolithic solutions. Moreover, although the performance of a single thread in the second experiment (Section 4.2) is inferior to the monolithic solution, it should be noted that this is at least partially the result of the cluster configuration selected to show the difference in behavior under increasing load. Moreover, the usage pattern was not optimal from the developed caching mechanism. This can also be observed in the double retrieval test depicted in Figure 9 or in the test presented in Section 4.3; the expected speed-up of the cache was noticeable, yet diminished, presumably by the DICOM transmission overhead.

Nevertheless, the performance for real-like traffic (Section 4.1, Section 4.2 and Section 4.3) is as expected for an online-grade archive. The instances are immediately available as required by DICOM standards (Section 4). The system can accommodate multiple clients and maintain similar per-thread performance. With the presented throughput, a typical MR or CT study (several dozens to a couple of hundred megabytes) can be downloaded in under two minutes, even in a single-threaded mode, and through direct DICOM-based retrieval, which should not be necessary for most scenarios. What is more, using S3 storage and microservice pattern in the cloud system permits scaling; multiple connections to high-latency storage service (such as AWS’s S3) can utilize more bandwidth and increase overall performance, despite the lower performance of a single thread. The same effect in the monolithic system can be accomplished by attaching the solution to a more efficient storage or index (i.e., scaling up).

On the other hand, it is worth pointing out that the total query time for unbound free-mode queries (without following the hierarchical DICOM data model and using optional keys) is short and aligns with expectations for an online grade DICOM archive. The query time for typical workloads following DICOM hierarchy (e.g., ‘get the data of a whole study’) is similar for monolithic and for cloud solutions (Section 4.2). What is more, the query times depicted in Figure 8 and given in Section 4.2 are also comparable, despite the fact that in the former case, the system was handling a repository that was larger by an order of magnitude, and optional keys were used in the query. The effect can presumably be attributed to the employment of a flat OpenSearch index. Based on this observation and the similar current outcomes of the production system, it is hoped that the performance will not deteriorate significantly with a constantly growing volume of data.

Unfortunately, for complex distributed systems, it is hardly ever possible to directly compare their performance. The results are affected by many factors simultaneously, for example, the hardware used, the algorithms employed, the type of network, and the operating system environment [56]. On the PACS level, different throughputs might be observed for different instance sizes and compression methods (see, for example, Section 4.3). For a reliable evaluation, one can compare the performance of the same software on different devices or run various programs in an identical environment [57]. On the other hand, despite using the monolithic reference of Dcm4chee in the test depicted in Section 4.2 to show the behavior difference under growing load in a similar cloud environment, we do not claim it under- or outperforms this solution on general terms, as neither the reference nor the presented environment was explicitly optimized for performance. Moreover, our test examines the core infrastructure, not the performance from the end-user point of view, which the bandwidth of the internet connection might limit. On the other hand, the bandwidth of said connection directly affects the user only for studies without local backup in the access device.

Similarly, comparing developed PACS with the other complex systems described in the literature based on numerical results might also be imperfect. Lebre et al. [41] repeated the storage and retrieval of a set of five files varying in size 10,000 times, achieving a wide range of speeds from 82 to 930 Mbps. Álvarez et al. [34] simply measured the combined caching and rendering times of several different images. Silva et al. [58] noted that in the Dicoogle environment, the time taken to retrieve query results is linear with regard to the collection size. For example, querying and retrieving 1500 instances from a database of 50,000 took 3500 ms (approximately). On the other hand, Valente et al. [33] chose a different way of evaluating the measurement, as they measured the overall time of data fetching (querying, downloading, and converting to the Portable Network Graphics (PNG) representation). Ribeiro et al. [39] implemented a custom load balancer in front of a distributed PACS, and their measurements of transmission time significantly depended on the actual network architecture and the number of parallel connections.

With time, a standard evaluation benchmark for PACS could be designed to allow for a more straightforward comparison. For example, the traffic pattern provided in [55] could be included to generate a similar data stream for comparing different systems. The synthetic tests presented in Section 4.2 or Section 4.3 are easy to reproduce and could be used to benchmark selected usage scenarios of multi-thread retrieval.

Using standard Internet application-layer protocols (mainly HTTP) permits the application of state-of-the-art security mechanisms (TLS, security tokens, etc.), as well as transfer optimizations (e.g., concurrent transfers). Having so-called ‘eleven 9’s’ (99.999999999%) availability, guaranteed by AWS on S3 storage, images are assumably securely archived and accessible. The same security level can be achieved in an on-premise, ‘traditional’ PACS by introducing back-up policies and external storage or implementing grid architectures [17].

The presented system is not free of limitations. Only the local archive is available without an internet connection, and remote storage or interpretation of images is impossible. Moreover, migrating a cloud archive to another provider can be challenging due to the sheer volume of data. However, similar problems apply to all PACS systems [59]. The novel cloud system establishes a base for future extension. In particular, the ability to integrate profoundly with AI tools should be emphasized, as presented in a paper by Cohen and Sodickson [60]. Dedicated screening algorithms can process selected studies on the fly [61]. However, optimizing the processing routines needs to be considered in order to move resource-demanding tasks to a separate cluster segment.

## 6. Conclusions

In the paper, an original PACS architecture was introduced. Although similar solutions have already been described, this system—to the best of the authors’ knowledge—is the first one explicitly designed for cloud-native deployment. The system features an architecture consisting of microservices, which allows the system to be deployed on an arbitrary cloud service provider. Currently, the system is running in an AWS environment, but during development, it worked successfully in a custom, local continuous integration environment and different commercial cloud environments (Polcom Cloud). The system is expected to be robust to faults with shared-nothing architecture and no single point of failure. Essential features include multi-tenancy and full scalability. Containerization of all microservices simplifies their management in a production environment.

The presented architecture is the most suitable solution when the number of clients dynamically changes over time and the load is highly variable. The dynamic scaling of microservices allows for the system to maintain fast responsiveness. If the upload, download, and retrieval load were constant over time, the architecture would seem unnecessarily complex.

Currently the system is deployed in the production environment of Radpoint Ltd. (Katowice, Poland) and serves over four thousand of distributed DICOM nodes.

## Figures and Tables

**Table 1:** Comparison of various PACS systems.

First Author and Year	Description	Advantages	Drawbacks
Warnock 2007 [37]	Dcm4chee PACS	Well known, fully featured PACS; wide usage	Heavyweight, no efficient scalability in 2.x series
Valente 2012 [33]	REST front-end to Dcm4chee; proof of concept	Modern network technology	Solely front-end
Ribeiro 2012 [39]	Dcm4chee based peer-to-peer PACS architecture	Improved performance regarding the transfer rate of DICOM objects	Shared-everything architecture
Valente 2016 [40]	Dicoogle PACS	Support for plugins for storage and index	Lack of integration with electronic health record systems
Álvarez 2017 [34]	Proof of concept of a distributed “PACS-as-a-service”	Modular architecture: a client-side, a server and a storage	lack of DICOM communication
Jodogne 2018 [35]	Orthanc PACS	Modular, lightweight	Designed as front-end to PACS; performance decreases with amount of stored data
Lebre 2021 [41]	Dicoogle-driven proof of concept of peer-to-peer storage	Automatic data redundancy	Unknown performance in production environment
this work	Presented system	High scalability	Overly complex for on-premise development

**Table 2:** Cloud PACS state as of October, 2022.

Parameter	Value
Total storage	490 TB
Total studies	4 M
Total series	23.3 M
Instance count	1.35 G
Registered access devices	415
New data per month *	33 TB
Central node instances	6

* On average during the past 3 months, including pending migrations from on-premise solutions.

**Table 3:** Test load: imaging modalities.

No.	Modality		Count
1	CT	Computed tomography	37
2	DX	Digital radiography	29
3	CR	Computed radiography	27
4	MR	Magnetic resonance	21
5	MG	Mammography	2
6	XA	X-ray angiography	1

**Table 4:** Data retrieval test from studies of various sizes. A selected number of retrieving threads were started with a series of C-MOVE operations issued for each study. Retrieval was finished when the last operation of the final thread was completed. The range of obtained results (worst and best case) is shown.

**Four Retrieving Threads**			
**Size of**	**Instances**	**Seconds per**	**Throughput**
**Study (MB)**	**per Second**	**100 Instances**	**(Mbps)**
2	23–24	4.2–4.3	93–96
4	25	3.9–4.0	100–102
8	22–23	4.4–4.5	89–92
16	22–23	4.3–4.6	87–93
32	22–23	4.4–4.6	88–90
64	23–25	3.9–4.3	93–102
128	26	3.8–3.9	104–106
256	26–28	3.5–3.8	105–114
512	31	3.2	124–126
1024	28	3.6	112–113
**Eight Retrieving Threads**			
**Size of**	**Instances**	**Seconds per**	**Throughput**
**Study (MB)**	**per Second**	**100 Instances**	**(Mbps)**
2	48–67	1.5–2.1	193–269
4	57–73	1.4–1.8	229–293
8	48–68	1.5–2.1	194–273
16	49–65	1.5–2.0	197–262
32	48–61	1.7–2.1	192–243
64	45–57	1.7–2.2	181–230
128	51–53	1.9–2.0	204–213
256	48–51	2.0–2.1	194–205
512	48–54	1.8–2.1	193–218
1024	48	2.1–2.1	193–194
**Sixteen Retrieving Threads**			
**Size of**	**Instances**	**Seconds per**	**Throughput**
**Study (MB)**	**per Second**	**100 Instances**	**(Mbps)**
2	71–85	1.2–1.4	284–343
4	79–112	0.9–1.3	316–449
8	81–102	1.0–1.2	325–409
16	84–109	0.9–1.2	336–437
32	82–104	1.0–1.2	328–418
64	77–104	1.0–1.3	310–420
128	84–92	1.1–1.2	335–370
256	85–91	1.1–1.2	339–367
512	86–93	1.1–1.2	347–373
1024	81–88	1.1–1.2	325–353

## Data Availability

The traffic model used in tests presented in Section 4 is openly available in Mendeley Data at DOI: 10.17632/n8mssthhnm (version 1) accessed on 13 September 2022.

## Abbreviations

The following abbreviations are used in this manuscript:

ACR	American College of Radiology
AI	Artificial Intelligence
API	Application Programming Interface
CAD	Computer-Aided Diagnosis
DICOM	Digital Imaging and Communications in Medicine
EBS	Elastic Block Store
HTTP	Hypertext Transfer Protocol
JWT	JSON Web Token
LAN	Local Area Network
NEMA	National Electrical Manufacturers Association
PACS	Picture Archiving and Communication System
PDU	Protocol Data Unit
REST	Representational State Transfer
SSL	Secure Socket Layer
VNA	Vendor Neutral Archive
VPN	Virtual Private Network
WAN	Wide Area Network

## References

[1] Fennell N., Ralston M.D., Coleman R.M., Branstetter B.F. (2021). PACS and Other Image Management Systems. Practical Imaging Informatics: Foundations and Applications for Medical Imaging.

[2] Huang H. (2010). PACS and Imaging Informatics: Basic Principles and Applications.

[3] Armbrust L.J. (2009). PACS and Image Storage. Vet. Clin. N. Am.-Small.

[4] Agarwal T.K., Sanjeev (2012). Vendor Neutral Archive in PACS. Indian J. Radiol. Imaging.

[5] Bidgood W.D., Horii S.C. (1992). Introduction to the ACR-NEMA DICOM Standard. RadioGraphics.

[6] Pianykh O.S. (2012). Digital Imaging and Communications in Medicine (DICOM).

[7] Bergh B. (2006). Enterprise imaging and multi-departmental PACS. Eur. Radiol..

[8] Costa C., Silva A., Oliveira J.L. (2007). Current Perspectives on PACS and a Cardiology Case Study. Advanced Computational Intelligence Paradigms in Healthcare-2.

[9] Herrmann M.D., Clunie D.A., Fedorov A., Doyle S.W., Pieper S., Klepeis V., Le L.P., Mutter G.L., Milstone D.S., Schultz T.J. (2018). Implementing the DICOM Standard for Digital Pathology. J. Pathol. Inform..

[10] Duncan L.D., Gray K., Lewis J.M., Bell J.L., Bigge J., McKinney J.M. (2010). Clinical Integration of Picture Archiving and Communication Systems with Pathology and Hospital Information System in Oncology. Am. Surg..

[11] Rybak G., Strzecha K., Krakós M. (2022). A New Digital Platform for Collecting Measurement Data from the Novel Imaging Sensors in Urology. Sensors.

[12] Halford J.J., Clunie D.A., Brinkmann B.H., Krefting D., Rémi J., Rosenow F., Husain A., Fürbass F., Ehrenberg J.A., Winkler S. (2021). Standardization of neurophysiology signal data into the DICOM^®^ standard. Clin. Neurophysiol..

[13] Gupta Y., Costa C., Pinho E., Silva L.B. (2022). DICOMization of Proprietary Files Obtained from Confocal, Whole-Slide, and FIB-SEM Microscope Scanners. Sensors.

[14] Faggioni L., Neri E., Castellana C., Caramella D., Bartolozzi C. (2011). The Future of PACS in Healthcare Enterprises. Eur. J. Radiol..

[15] Cawthra J., Hodges B., Kuruvilla J., Littlefield K., Niemeyer B., Peloquin C., Wang S., Williams R., Zheng K. (2020). Securing Picture Archiving and Communication System (PACS) Cybersecurity for the Healthcare Sector.

[16] Cordero D., Barría C., Latifi S. (2021). Cybersecurity Analysis in Nodes That Work on the DICOM Protocol, a Case Study. ITNG 2021 18th International Conference on Information Technology-New Generations.

[17] Huang H.K., Zhang A., Liu B., Zhou Z., Documet J., King N., Chan L.W.C. (2005). Data grid for large-scale medical image archive and analysis. Proceedings of the 13th annual ACM international conference on Multimedia—MULTIMEDIA ’05.

[18] Kagadis G.C., Kloukinas C., Moore K., Philbin J., Papadimitroulas P., Alexakos C., Nagy P.G., Visvikis D., Hendee W.R. (2013). Cloud computing in medical imaging. Med. Phys..

[19] Khajouei R., Jahromi M.E., Ameri A. (2019). Challenges of Implementing Picture Archiving and Communication System in Multiple Hospitals: Perspectives of Involved Staff and Users. J. Med. Syst..

[20] Fridell K., Aspelin P., Edgren L., Lindsköld L., Lundberg N. (2009). PACS influence the radiographer’s work. Radiography.

[21] Yu T.Y., Ho H.H. (2008). The design and development of a physician-oriented PACS for the enhancement of e-hospital facilities. Int. J. Med. Inform..

[22] European Society of Radiology (ESR) (2013). Organisation and practice of radiological ultrasound in Europe: A survey by the ESR Working Group on Ultrasound. Insights Imaging.

[23] European Society of Radiology (ESR) (2020). Position statement and best practice recommendations on the imaging use of ultrasound from the European Society of Radiology ultrasound subcommittee. Insights Imaging.

[24] Ratib O. (2011). From PACS to the Clouds. Eur. J. Radiol..

[25] Philbin J., Prior F., Nagy P. (2011). Will the Next Generation of PACS Be Sitting on a Cloud?. J. Digit. Imaging.

[26] Kratzke N., Quint P.C. (2017). Understanding cloud-native applications after 10 years of cloud computing—A systematic mapping study. J. Syst. Softw..

[27] Langer S.G. (2011). Challenges for Data Storage in Medical Imaging Research. J. Digit. Imaging.

[28] Shini S.G., Thomas T., Chithraranjan K. (2012). Cloud Based Medical Image Exchange—Security Challenges. Procedia Eng..

[29] Savaris A., Gimenes Marquez Filho A.A., Rodrigues Pires de Mello R., Colonetti G.B., Von Wangenheim A., Krechel D. Integrating a PACS Network to a Statewide Telemedicine System: A Case Study of the Santa Catarina State Integrated Telemedicine and Telehealth System. Proceedings of the 2017 IEEE 30th International Symposium on Computer-Based Medical Systems (CBMS).

[30] Berkowitz S.J., Wei J.L., Halabi S. (2018). Migrating to the Modern PACS: Challenges and Opportunities. RadioGraphics.

[31] Dikici E., Bigelow M., Prevedello L.M., White R.D., Erdal B.S. (2020). Integrating AI into radiology workflow: Levels of research, production, and feedback maturity. J. Med. Imag..

[32] Horii S.C., Behlen F.M., Branstetter B.F. (2021). PACS Readiness and PACS Migration. Practical Imaging Informatics: Foundations and Applications for Medical Imaging.

[33] Valente F., Viana-Ferreira C., Costa C., Oliveira J.L. (2012). A RESTful Image Gateway for Multiple Medical Image Repositories. IEEE Ttans. Inf. Technol. Biomed..

[34] Álvarez R., Legarreta J.H., Kabongo L., Epelde G., Macía I. (2017). Towards a Deconstructed PACS-as-a-Service System. International Conference on Innovation in Medicine and Healthcare.

[35] Jodogne S. (2018). The Orthanc Ecosystem for Medical Imaging. J. Digit. Imaging.

[36] Sohn J.H., Chillakuru Y.R., Lee S., Lee A.Y., Kelil T., Hess C.P., Seo Y., Vu T., Joe B.N. (2020). An Open-Source, Vender Agnostic Hardware and Software Pipeline for Integration of Artificial Intelligence in Radiology Workflow. J. Digit. Imaging.

[37] Warnock M.J., Toland C., Evans D., Wallace B., Nagy P. (2007). Benefits of Using the DCM4CHE DICOM Archive. J. Digit. Imaging.

[38] Costa C., Ferreira C., Bastião L., Ribeiro L., Silva A., Oliveira J.L. (2011). Dicoogle—An Open Source Peer-to-Peer PACS. J. Digit. Imaging.

[39] Ribeiro L.S., Costa C., Oliveira J.L. (2012). Clustering of Distinct PACS Archives Using a Cooperative Peer-to-Peer Network. Comput. Meth. Prog. Biomed..

[40] Valente F., Silva L.A.B., Godinho T.M., Costa C. (2016). Anatomy of an Extensible Open Source PACS. J. Digit. Imaging.

[41] Lebre R., Silva L.B., Costa C. Decentralizing the Storage of a DICOM Compliant PACS. Proceedings of the 2021 IEEE International Conference on Bioinformatics and Biomedicine (BIBM).

[42] Lebre R., Pinho E., Silva J.M., Costa C. Dicoogle Framework for Medical Imaging Teaching and Research. Proceedings of the 2020 IEEE Symposium on Computers and Communications (ISCC).

[43] Faggioni L., Neri E., Cerri F., Turini F., Bartolozzi C. (2011). Integrating Image Processing in PACS. Eur. J. Radiol..

[44] Pietka E., Kawa J., Spinczyk D., Badura P., Wieclawek W., Czajkowska J., Rudzki M. (2011). Role of radiologists in CAD life-cycle. Eur. J. Radiol..

[45] Lui Y.W., Geras K., Block K.T., Parente M., Hood J., Recht M.P. (2020). How to Implement AI in the Clinical Enterprise: Opportunities and Lessons Learned. J. Am. Coll. Radiol..

[46] Kotter E., Ranschaert E. (2020). Challenges and solutions for introducing artificial intelligence (AI) in daily clinical workflow. Eur. Radiol..

[47] Ranschaert E., Topff L., Pianykh O. (2021). Optimization of Radiology Workflow with Artificial Intelligence. Radiol. Clin. N. Am..

[48] Jiang L., Wu Z., Xu X., Zhan Y., Jin X., Wang L., Qiu Y. (2021). Opportunities and challenges of artificial intelligence in the medical field: Current application, emerging problems, and problem-solving strategies. J. Int. Med. Res..

[49] Gannon D., Barga R., Sundaresan N. (2017). Cloud-Native Applications. IEEE Cloud Comput..

[50] Jamshidi P., Pahl C., Mendonca N.C., Lewis J., Tilkov S. (2018). Microservices: The Journey So Far and Challenges Ahead. IEEE Softw..

[51] Plecinski P., Bokla N., Klymkovych T., Melnyk M., Zabierowski W. (2022). Comparison of Representative Microservices Technologies in Terms of Performance for Use for Projects Based on Sensor Networks. Sensors.

[52] Bushong V., Abdelfattah A.S., Maruf A.A., Das D., Lehman A., Jaroszewski E., Coffey M., Cerny T., Frajtak K., Tisnovsky P. (2021). On Microservice Analysis and Architecture Evolution: A Systematic Mapping Study. Appl. Sci..

[53] Szalay M., Mátray P., Toka L. (2021). State Management for Cloud-Native Applications. Electronics.

[54] Linthicum D.S. (2017). Cloud-Native Applications and Cloud Migration: The Good, the Bad, and the Points Between. IEEE Cloud Comput..

[55] Kawa J. DICOM Traffic Record from Different Medical Imaging Devices. Mendeley Data. https://data.mendeley.com/datasets/n8mssthhnm.

[56] Alzakholi O., Haji L., Shukur H., Zebari R., Abas S., Sadeeq M. (2020). Comparison Among Cloud Technologies and Cloud Performance. J. Appl. Sci. Technol. Trends.

[57] Pyciński B., Kawa J., Bożek P., Smoliński M., Bieńkowska M. (2019). Performance of Medical Image Transfer in High Bandwidth Networks. Innovations in Biomedical Engineering.

[58] Silva L.A.B., Beroud L., Costa C., Oliveira J.L. Medical Imaging Archiving: A Comparison between Several NoSQL Solutions. Proceedings of the IEEE-EMBS International Conference on Biomedical and Health Informatics (BHI).

[59] Van Ooijen P.M.A., Aryanto K.Y., Broekema A., Horii S. (2015). DICOM Data Migration for PACS Transition: Procedure and Pitfalls. Int. J. Comput. Assist. Rad..

[60] Cohen R.Y., Sodickson A.D. (2021). An Orchestration Platform that Puts Radiologists in the Driver’s Seat of AI Innovation: A Methodological Approach. arXiv.

[61] Tang A., Tam R., Cadrin-Chênevert A., Guest W., Chong J., Barfett J., Chepelev L., Cairns R., Mitchell J.R., Cicero M.D. (2018). Canadian Association of Radiologists White Paper on Artificial Intelligence in Radiology. Can. Assoc. Radiol. J..

